# A case of IgG4-related ophthalmic disease after SARS-CoV-2 vaccination: case report and literature review

**DOI:** 10.3389/fimmu.2024.1303589

**Published:** 2024-02-22

**Authors:** Peixuan Zhang, Qian Wu, Xiao Xu, Minliang Chen

**Affiliations:** ^1^ Department of Burns and Plastic Surgery, The Forth Medical Center of Chinese PLA General Hospital, Beijing, China; ^2^ Department of Medical Service, The Sixth Medical Center of Chinese PLA General Hospital, Beijing, China; ^3^ Senior Department of Ophthalmology, The Third Medical Center of Chinese PLA General Hospital, Beijing, China

**Keywords:** autoimmunity, case report, COVID-19, IgG4, IgG4-related ophthalmic disease, SARS-CoV-2

## Abstract

Coronavirus disease 19 (COVID-19) caused by severe acute respiratory syndrome coronavirus 2 (SARS-CoV-2) is affecting the world with a surge in cases. A variety of autoimmune diseases occur after SARS-CoV-2 infection or vaccination, of which IgG4-related disease (IgG4-RD) is an important type. IgG4-RD can involve multiple organs of the body. The ocular manifestation of IgG4-RD is called IgG4-related ophthalmic disease (IgG4-ROD). We herein report a patient diagnosed with IgG4-ROD. The patient developed ptosis and vision loss after SARS-CoV-2 vaccination, and the symptoms worsened after SARS-CoV-2 infection. After excluding other diseases like myasthenia gravis and Eaton-Lambert syndrome that may cause ptosis, the diagnosis of IgG4-ROD was confirmed by pathological examination. We discussed the predisposing factors, diagnosis and treatment of this patient to provide a more empirical and theoretical basis for clinical diagnosis and treatment. We conducted a literature review of previously reported cases of IgG4-RD following SARS-CoV-2 infection or vaccination. We retrieved a total of 9 cases, of which 5 developed symptoms after vaccination and 4 after infection. Demographic and clinical characteristics were summarized. In conclusion, our case represents the first case of proven IgG4-ROD after COVID-19 vaccination. We believe that IgG4-ROD and SARS-CoV-2 infection or vaccination are closely related, and the immune system disorder caused by SARS-CoV-2 infection or vaccination may be a key factor in the pathogenesis of IgG4-RD. But for now, there is no direct evidence that there is a causal relationship between SARS-CoV-2 infection or vaccination and IgG4-ROD, which still needs more research and exploration to confirm.

## 1 Introduction

Severe acute respiratory syndrome coronavirus 2 (SARS-CoV-2) is a highly contagious coronavirus, which causes an acute respiratory disease called coronavirus disease 19 (COVID-19) (1). COVID-19 does not only cause inflammatory symptoms such as fever and cough but also has a huge impact on the immune system. Vaccination is one of the most effective interventions to substantially reduce severe disease and death due to SARS-CoV-2 infection. At present, there is some evidence that a variety of different autoantibodies appear in patients after SARS-CoV-2 infection or vaccination, and a number of them develop autoimmune diseases, such as autoimmune hemolytic anemia, Guillain‐Barre syndrome, and so on (2–4).

IgG4-related disease (IgG4-RD) is an autoimmune disease with undetermined pathogenesis, in which multiple organs and tissues can be involved (5). IgG4-RD is characterized by infiltration of IgG4-immunopositive plasmacytes and elevated serum IgG4 concentration accompanied by enlargement and mass in various organs (6). Several previous case reports had reported the development of IgG4-RD after SARS-CoV-2 infection or vaccination, patients showed different symptoms. The ocular manifestation of IgG4-RD is called IgG4-related ophthalmic disease (IgG4-ROD). IgG4-ROD most commonly affects the lacrimal gland, but orbital soft tissue, orbital nerves, sclera, choroid, and orbital adnexa can also be involved. It presents insidiously, with symptoms of painless proptosis, eyelid swelling, decreased vision with tearing, pain, redness, and photopsia (7). IgG4-ROD may involve a single structure or multiple structures within the orbit, and may also exhibit bilateral disease (8).

There are currently no internationally standardized diagnostic criteria for IgG4-ROD. The most recent diagnostic criteria proposed by Japanese researchers in 2015 are: (1) Imaging studies show enlargement of the lacrimal gland, trigeminal nerve, or extraocular muscle as well as masses, enlargement, or hypertrophic lesions in various ophthalmic tissues; (2) Histopathologic examination shows marked lymphocyte and plasmacyte infiltration, and sometimes fibrosis. A germinal center is frequently observed. IgG4^+^ plasmacytes are found and satisfy the following criteria: ratio of IgG4+ cells to IgG4^+^ cells of 40% or above, or more than 50 IgG4^+^ cells per high-power field (×400); (3) Blood test shows elevated serum IgG4 (≥135 mg/dl). Diagnosis is classified as ‘‘definitive’’ when (1), (2), and (3) are satisfied; ‘‘probable’’ when (1) and (2) are satisfied; and ‘‘possible’’ when (1) and (3) are satisfied (9). In terms of treatment, intravenous glucocorticoid therapy is the first-line treatment for IgG4-ROD, which is effective and well-tolerated (10). Rituximab is also a treatment option for some refractory patients (11).

We herein report a patient diagnosed with IgG4-ROD. The patient developed ptosis and vision loss after SARS-CoV-2 vaccination, and the symptoms worsened after SARS-CoV-2 infection. We admitted her to the ophthalmic ward of the Third Medical Center of Chinese PLA General Hospital. We recorded the changes in her condition and reviewed the previous literature.

## 2 Case description

The patient, a previously healthy 22-year-old female, received an inactivated COVID-19 vaccine in May 2021 and reported fever within the next few days. After a month, the patient noticed a slight ptosis in the left eye and a mild loss of vision in the right eye, and these symptoms gradually worsened. In January 2022, the patient felt that the above symptoms had progressed to the point where they were affecting her life and went to the local hospital. Orbital CT showed soft tissue swelling in the left lacrimal gland region. The funduscopy examination revealed macular edema in the right eye. Fundus fluorescein angiography showed tortuous fundus veins in the right eye, anastomosed vessels in the optic disc and retina, and extensive peripheral retinal vessel dilation and leakage. During hospitalization, compound anisodine was injected into the left superficial temporal artery, together with oral mecobalamin, Mongolian medicine, acupuncture, and electromagnetic therapy, the patient felt that the symptoms were worse than before.

**Figure 1:**
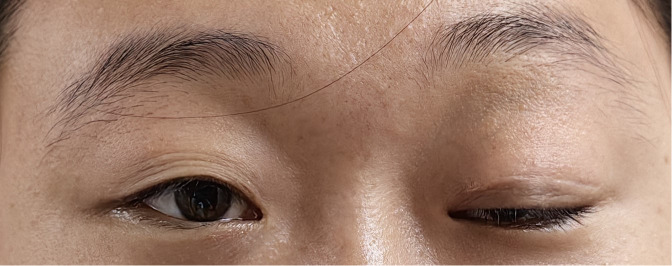
Photograph of the patient on admission.

**Figure 2:**
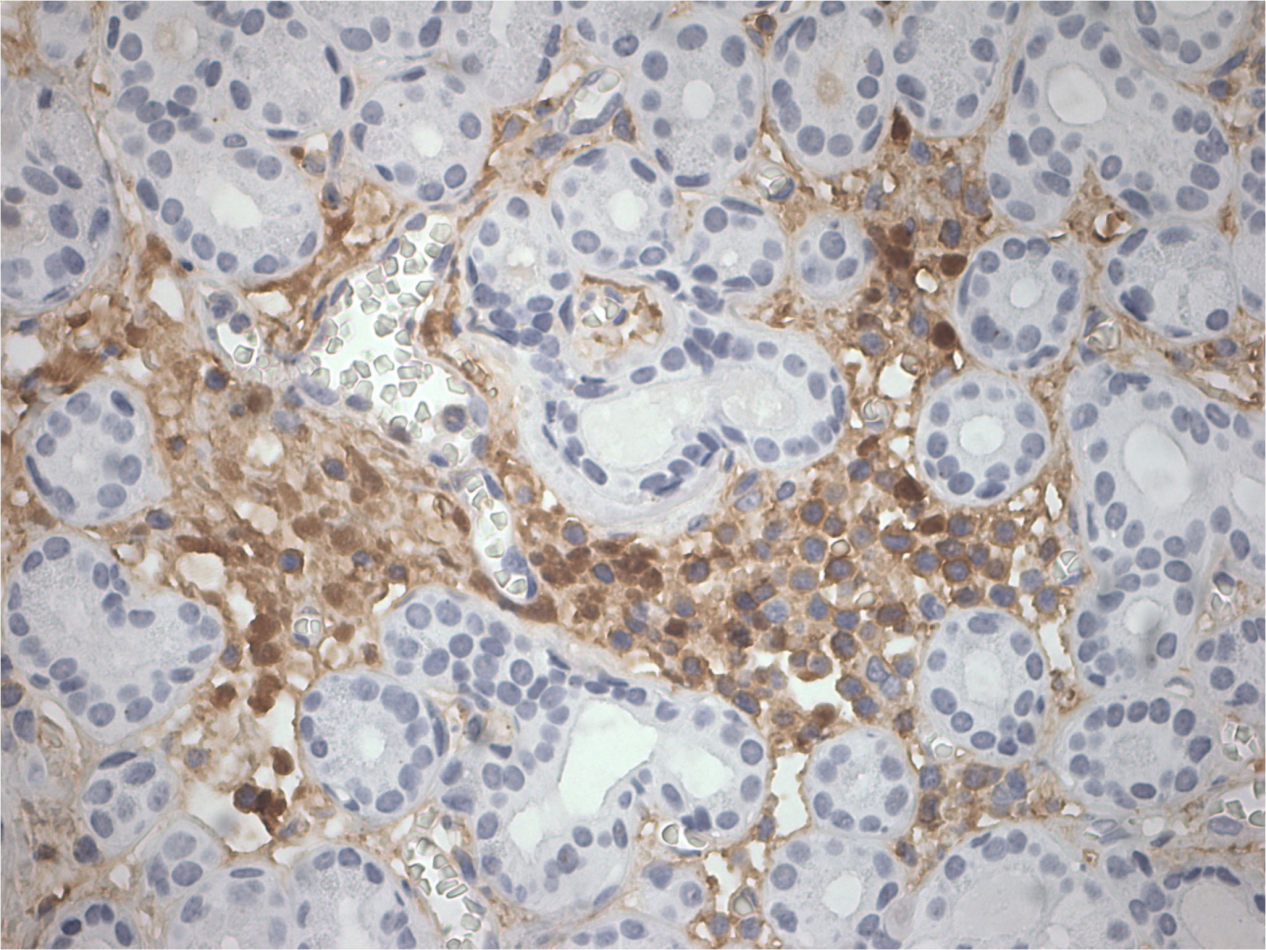
IgG4^+^ cells (×400).

The patient was admitted to our hospital in August 2022. After admission, the visual acuity of the right eye was 0.3 and the left eye was 0.8, the ptosis of the left eye covered the pupil more than 1/2 (**Figure 1**). Initially, we considered a diagnosis of myasthenia gravis and central retinal vein occlusion in the right eye. Serial tests were performed to identify the differential diagnosis. Electromyography showed that repetitive nerve electrical stimulation did not show characteristic changes. The fatigue test and the neostigmine test were negative. Serum specific antibodies for neuromuscular disease were all negative. The diagnosis of neuromuscular diseases like myasthenia gravis and Eaton-Lambert syndrome can be ruled out. However, we noted that serum IgG4 was 280 mg/dl and ocular ultrasound suggested uneven enlargement of the left lacrimal gland and fullness of the right lacrimal gland. To investigate the presence of neuropathy, an MRI of the head was performed, which revealed the optic nerve in the right orbit was slightly thinner than normal with hyperintensity on T2WI and abnormal enhancement of the optic nerve and its surrounding sheath. On August 23, 2022, a biopsy of the left lacrimal gland, orbital septal tissue, and orbital fat was performed by us. The result of histopathology was inflammatory pseudotumor. Immunohistochemical showed that the ratio of IgG4^+^ plasmacytes to IgG^+^ plasmacytes was more than 40% and more than 50 IgG4^+^ cells per high-power field (×400) (**Figure 2**). We performed a staining of the CD4 cells (**Supplementary Figure 1**) and CD8 (**Supplementary Figure 2**) cells in the biopsy. This patient satisfied diagnostic criteria (1), (2), and (3) for a definite diagnosis of IgG4-ROD. After diagnosis, the patient was given high-dose intravenous methylprednisolone pulse therapy and tacrolimus. After five days of treatment, the patient reported opening the eye more easily, but there was no significant change in the ptosis and vision loss. The patient asked to be discharged. After discharge, the patient continued to take methylprednisolone orally until November 2022 and tacrolimus orally until August 2023.

**Figure 3:**
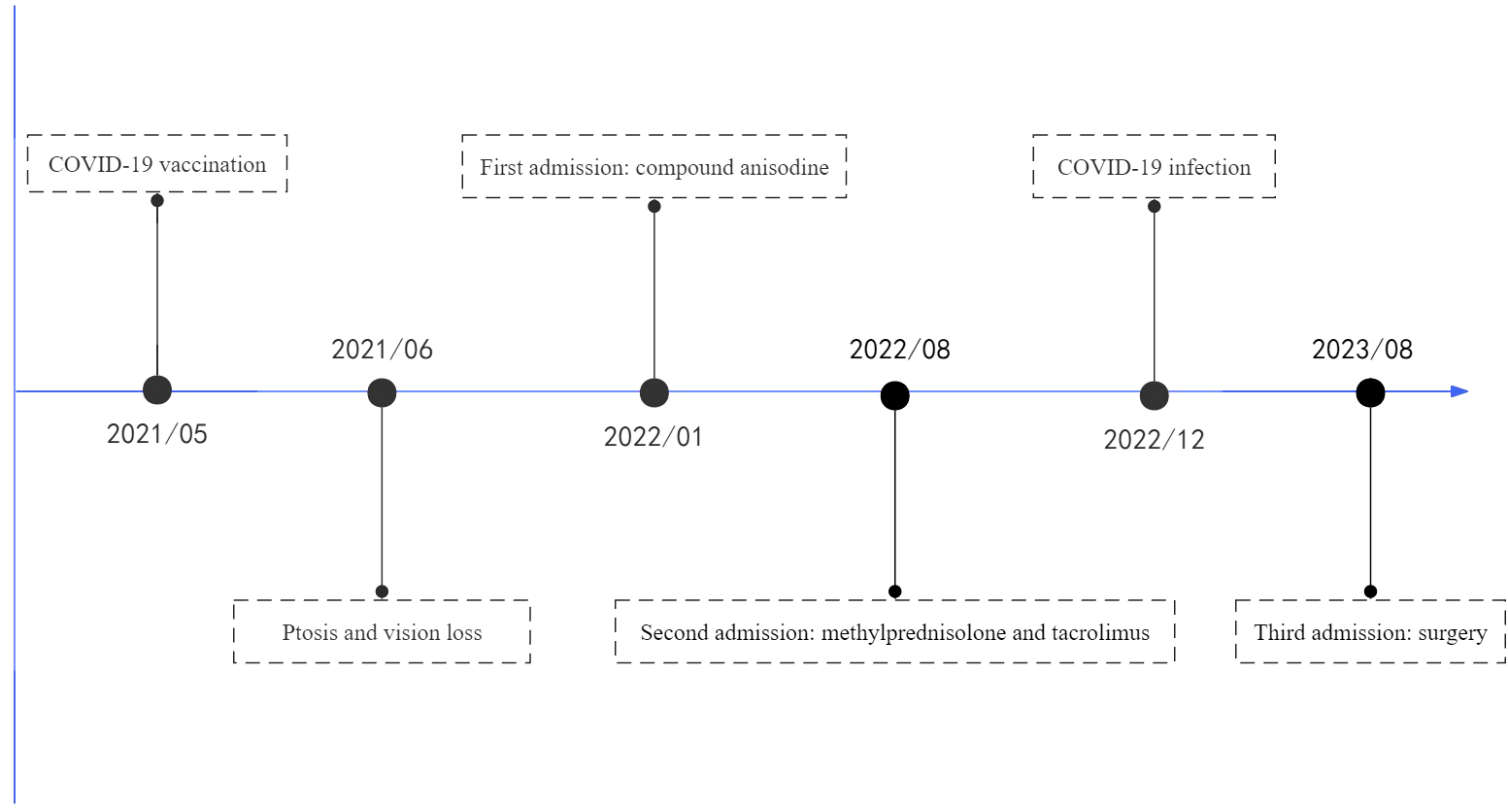
Timeline of treatment.

Unfortunately, the patient was infected with COVID-19 in December 2022. Although the patient remained on oral tacrolimus, the symptoms of ptosis in her left eye and vision loss in her right eye worsened after infection, and she was readmitted in August 2023. The visual acuity of the right eye was 0.15 and the left eye was 1.0, the ptosis of the left eye covered the entire pupil. The serum IgG4 was 240 mg/dl. Given the poor response to steroids therapy and the patient’s request for surgery to improve ptosis, we performed ptosis correction of left eye and bilateral double eyelid plasty on August 15, 2023 (**Figure 3**). During the ptosis correction, we performed a pathologic examination of a piece of the levator palpebrae superioris muscle, which showed fibrosis. The patient recovered well after the operation, and the eyelid margins of both eyes were symmetrical.

## 3 Discussion

The SARS-CoV-2, responsible for COVID-19, has spread across the world. The clinical manifestations of COVID-19 vary greatly, but it is worth noting that some patients can develop long-term complications, including the respiratory system, cardiovascular system, and nervous system (12). There is growing evidence that COVID-19 can cause autoimmune diseases. It has been suggested that common pathogenetic and clinical-radiological aspects between hyperinflammatory diseases and COVID-19 may indicate that SARS-CoV-2 may be a trigger for rapid autoimmune or autoinflammatory dysregulation (13). Several mechanisms, including molecular mimicry, bystander activation of T-cells, and epitope spreading, as for explaining how viral infection can trigger a response leading to autoimmune disease (14).

As the most important means to prevent SARS-CoV-2 infection, the COVID-19 vaccine has been widely used worldwide. Although it is well tolerated by most people, adverse reactions after vaccination should not be ignored. Vaccines induce adaptive immune responses to exert their protective effects, but may induce a hyperinflammatory state. A few cases of new or recurrent autoimmune disease have been reported after COVID-19 vaccination. Pichi et al. reported nine eyes presenting with ocular adverse events after the first inoculation of inactivated COVID-19 vaccine (15). It is assumed that COVID-19 vaccine can trigger autoimmunity through molecular mimicry, the production of particular autoantibodies, and the role of certain vaccine adjuvants (16).

We conducted a literature review of previously reported cases of IgG4-RD following SARS-CoV-2 infection or vaccination (**Table 1**) (17–25). We retrieved a total of 9 cases, of which 5 developed symptoms after vaccination and 4 after infection. Of the vaccinated patients, 4 received mRNA vaccine, including 3 who received the mRNA BNT162b2 vaccine. The interval between the onset of IgG4-RD symptoms and SARS-CoV-2 infection or vaccination ranged from one day to three and a half months. IgG4-RD can affect a wide range of organs, and patients can be clinically manifested as hepatopathy, lung disease, pancreatitis, sialadenitis, rhinosinusitis, lymph node enlargement, and parotid enlargement. After surgery and prednisone therapy, most patients’ symptoms were relieved or even recovered.

**Table 1:** Demographic and clinical characteristics of previously reported IgG4-related disease following COVID-19.

Author	Year	Age	Sex	SARS-CoV-2	Vaccine Type	Interval time	Clinical Manifestations	Treatment	Outcome
Rodrigues	2022	66	Male	Vaccination	mRNA BNT162b2	2 weeks	Autoimmune pancreatitis	Prednisone	Recovered
Patel	2022	63	Male	Vaccination	mRNA	2 months	Autoimmune pancreatitis	Prednisone	Recovered
Tasnim	2022	71	Male	Vaccination	mRNA BNT162b2	2 weeks	Lung disease	Thoracoscopic drainage Decortication	Further treatment
Aochi	2022	78	Female	Vaccination	mRNA BNT162b2	2 weeks	Sialadenitis Autoimmune pancreatitis	Prednisone	Recovered
Kuno	2023	84	Female	Vaccination	/	1 day	Hepatopathy	No treatment	Recovered
Roncati	2020	67	Male	Infection	/	3.5 months	Subglottic stenosis	Surgery	Symptom relief
Harb	2021	70	Male	Infection	/	1 week	Rhinosinusitis	Prednisone Surgery	Recovered
Xu	2023	50	Male	Infection	/	2 months	Pleura lymph node enlargement	Not mentioned	Not mentioned
Gurunathan	2023	36	Female	Infection	/	3 months	Sinusitis Parotid enlargement	Prednisolone Mycophenolate mofetil Rituximab	Symptoms controlled

COVID-19, coronavirus disease 19; SARS-CoV-2, severe acute respiratory syndrome coronavirus 2.

In our literature review, we found four patients who developed IgG4-RD after mRNA vaccination, whereas the patient we report here received an inactivated vaccine. Based on the available evidence, it is not clear whether different COVID-19 vaccine types have an effect on IgG4-RD pathogenesis. The mRNA in mRNA vaccines can present as either an antigen or an adjuvant, and can be recognized and bound by endosomal Toll-like receptors (TLRs) and inflammasomes to induce inflammation and immune responses (26). It has been found that TLRs play an important role in the pathogenesis of IgG4-RD. M2 macrophages expressing TLR7 in diseased organs recognize the COVID-19 mRNA or their own RNAs released by stressed or injured cells and promote the production of a variety of fibrotic cytokines through TLR7/IRAK4/NF-κB signaling, leading to severe fibrosis in diseased organs (27). Furthermore, after injection of the COVID-19 mRNA vaccine, the mRNA enters the muscle cells and the ribosomes perform cellular translation to produce the spike protein (16). Multiple amino acid fragments on the SARS-CoV-2 spike protein have been identified as molecular mimics of human proteins, which can induce the immune system to produce autoantibodies (28). Although the inactivated COVID-19 vaccine cannot be translated *in vivo* like the mRNA vaccine, it carries the spike protein itself. When individuals are exposed to antigens for prolonged period of time, the humoral immune response eventually switches to IgG4. The proportion of IgG4 in immunoglobulin would increase significantly after multiple vaccination or breakthrough infection with SARS-CoV-2 (29). Recently revealed a novel subset of IL-10^+^LAG3^+^ T follicular cells infiltrating the affected organs of IgG4-RD patients, it may play an important role in driving IgG4 class switching (30). CD4^+^ helper T cells are the most abundant cells in tissues affected by IgG4-RD and are thought to be the driver of IgG4-RD pathogenesis (31). Under some unknown conditions, CD4^+^ helper T cells are activated in response to long-standing COVID-19 spike protein or other antigenic stimulation, thereby promoting the differentiation of IL-10^+^LAG3^+^ T follicular cells, driving IgG4 class switching.

IgG4-RD is an immune-mediated fibro-inflammatory disease, which usually leads to tumefactive lesions and fibrosis (32, 33). We considered the following two possible causes of this patient’s ptosis: the first was the compression of the oculomotor nerve by the swelling of the orbital soft tissue caused by IgG4-ROD, and the second was the fibrosis of the levator muscle of the upper eyelid caused by IgG4-ROD. It has been suggested that serum IgG4 levels are useful in assessing dynamic disease severity and monitoring relapse (34). This is essential for physicians to treat IgG4-RD clinically. Kobak et al. reported a patient with IgG4-RD, who had a good response to treatment, but eventually causing IgG4-RD recurrence after infection with SARS-CoV-2 (35). Relapse of IgG4-RD is not only secondary to COVID-19 but may also be secondary to mRNA vaccination (36). Egashira et al. reported the development of cerebral venous sinus thrombosis in a patient with IgG4-ROD during recovery from COVID-19 (37). Cerebral venous sinus thrombosis is an important complication of COVID-19, which suggests that IgG4-ROD patients are more likely to have complications of COVID-19. A prospective study reported that serum IgG4 levels may predict the prognosis of COVID-19, a concentration of serum IgG4>700 mg/dl and an IgG4/IgG1 ratio>0.05 were associated with a significantly increased mortality at 30 days (38).

As to the cause of this patient’s vision loss in the right eye, the central retinal vein occlusion was confirmed on examination. Ocular vascular embolism after COVID-19 vaccination is a rare complication (39). The exact mechanisms have yet to be ascertained. Leung et al. raised three possible mechanisms: vaccine-induced immune thrombotic thrombocytopenia, COVID-19 vaccine as a trigger in homocysteinaemia, and retinal vasculitis (40). There are a few reports of vision loss caused by central retinal vein thrombosis after COVID-19 vaccination, and vision can return to normal after timely treatment (41, 42). However, a self-controlled case series found no evidence of an association between retinal vein thrombosis and COVID-19 vaccination (43).

In conclusion, our case represents the first case of proven IgG4-ROD after COVID-19 vaccination. We believe that IgG4-ROD and SARS-CoV-2 infection or vaccination are closely related, and the immune system disorder caused by SARS-CoV-2 infection or vaccination may be a key factor in the pathogenesis of IgG4-RD. But for now, there is no direct evidence that there is a causal relationship between SARS-CoV-2 infection or vaccination and IgG4-ROD, which still needs more research and exploration to confirm.

## Data availability statement

The original contributions presented in the study are included in the article/**Supplementary Material**. Further inquiries can be directed to the corresponding authors.

## Ethics statement

Written informed consent was obtained from the individual(s) for the publication of any potentially identifiable images or data included in this article.

## Author contributions

PZ: Writing – original draft. QWu: Writing – review & editing. XX: Writing – review & editing. MC: Writing – review & editing.

## Supplementary material

Supplementary Figure 1 CD4 cells (×100).

Supplementary Figure 2 CD8 cells (×100).
