# Supplementary figures and images for: A case of IgG4-related ophthalmic disease after SARS-CoV-2 vaccination: case report and literature review
Source: Front Immunol. 2024 Feb 22;15:1303589. doi: 10.3389/fimmu.2024.1303589 (PMC10917890; doi:10.3389/fimmu.2024.1303589)

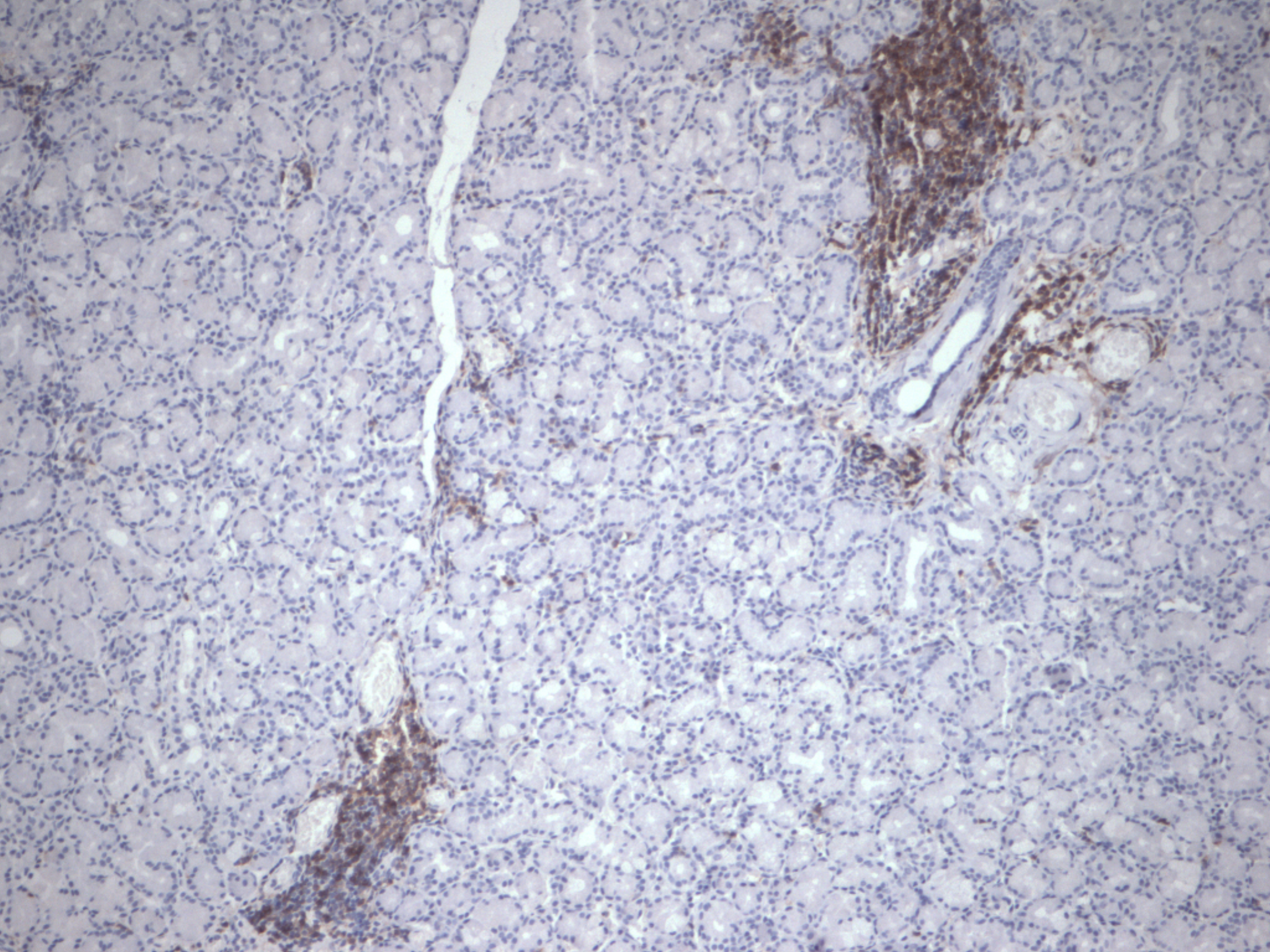

Supplement: Supplementary Figure 1 — CD4 cells (×100). [file Image_1.tif]

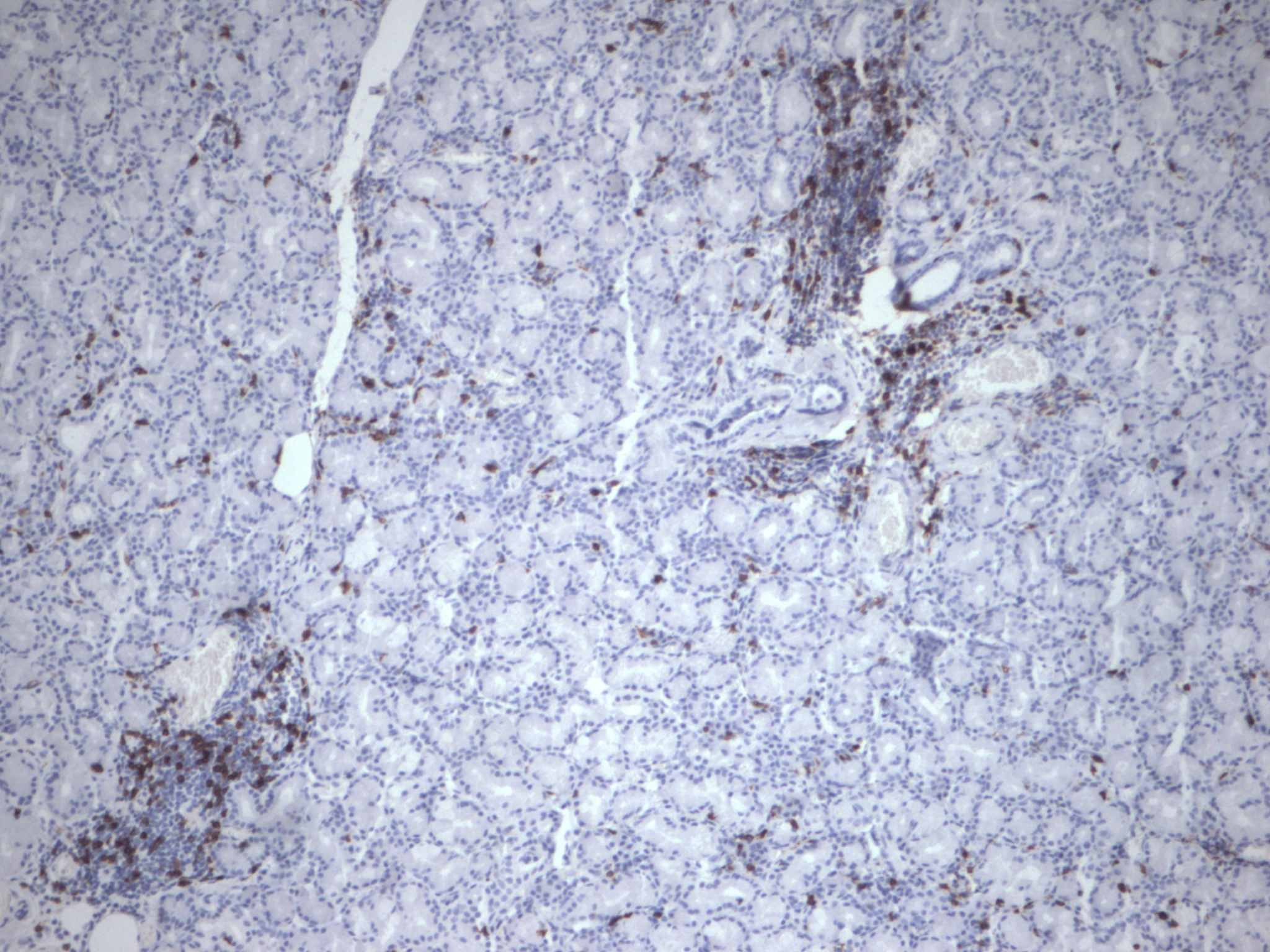

Supplement: Supplementary Figure 2 — CD8 cells (×100). [file Image_2.tif]
